# Analytics of self-regulated learning scaffolding: effects on learning processes

**DOI:** 10.3389/fpsyg.2023.1206696

**Published:** 2023-08-03

**Authors:** Tongguang Li, Yizhou Fan, Yuanru Tan, Yeyu Wang, Shaveen Singh, Xinyu Li, Mladen Raković, Joep van der Graaf, Lyn Lim, Binrui Yang, Inge Molenaar, Maria Bannert, Johanna Moore, Zachari Swiecki, Yi-Shan Tsai, David Williamson Shaffer, Dragan Gašević

**Affiliations:** ^1^Faculty of Information Technology, Monash University, Clayton, VIC, Australia; ^2^Graduate School of Education, Peking University, Beijing, China; ^3^School of Informatics, University of Edinburgh, Edinburgh, United Kingdom; ^4^Department of Educational Psychology, University of Wisconsin-Madison, Madison, WI, United States; ^5^Behavioural Science Institute, Radboud University, Nijmegen, Gelderland, Netherlands; ^6^School of Social Sciences and Technology, Technical University of Munich, Munich, Bavaria, Germany

**Keywords:** self-regulated learning, SRL process, SRL scaffolding, ordered network analysis, segmentation analysis

## Abstract

Self-regulated learning (SRL) is the ability to regulate cognitive, metacognitive, motivational, and emotional states while learning and is posited to be a strong predictor of academic success. It is therefore important to provide learners with effective instructions to promote more meaningful and effective SRL processes. One way to implement SRL instructions is through providing real-time SRL scaffolding while learners engage with a task. However, previous studies have tended to focus on fixed scaffolding rather than adaptive scaffolding that is tailored to student actions. Studies that have investigated adaptive scaffolding have not adequately distinguished between the effects of adaptive and fixed scaffolding compared to a control condition. Moreover, previous studies have tended to investigate the effects of scaffolding at the task level rather than shorter time segments—obscuring the impact of individual scaffolds on SRL processes. To address these gaps, we (a) collected trace data about student activities while working on a multi-source writing task and (b) analyzed these data using a cutting-edge learning analytic technique— ordered network analysis (ONA)—to model, visualize, and explain how learners' SRL processes changed in relation to the scaffolds. At the task level, our results suggest that learners who received adaptive scaffolding have significantly different patterns of SRL processes compared to the fixed scaffolding and control conditions. While not significantly different, our results at the task segment level suggest that adaptive scaffolding is associated with earlier engagement in SRL processes. At both the task level and task segment level, those who received adaptive scaffolding, compared to the other conditions, exhibited more task-guided learning processes such as referring to task instructions and rubrics in relation to their reading and writing. This study not only deepens our understanding of the effects of scaffolding at different levels of analysis but also demonstrates the use of a contemporary learning analytic technique for evaluating the effects of different kinds of scaffolding on learners' SRL processes.

## 1. Introduction

### 1.1. Self-regulated learning and scaffolding

Self-regulated learning (SRL) encompasses multiple cognitive, motivational, and emotional aspects of learning and has been thoroughly researched and integrated into education (Panadero, 2017). Contrary to views of value achievement, SRL emphasizes the mechanisms by which learners actively control and adjust their learning in response to varying educational contexts (Zimmerman, 1986). Various SRL models have been proposed throughout the years. One such model is the COPES model by Winne and Hadwin (1998), which describes four learning phases—task definition, goal setting and planning, enactment, and adaptation—coupled with five learning facets – condition, operation, product, evaluation, and standard (the COPES model). In this model, learners' strategies are influenced by internal and external conditions to manage learning information and, in turn, produce a learning product. This product is then evaluated against internal and external standards to facilitate learning adaptation. Similarly, Bannert (2007) proposed a comprehensive SRL framework for hypermedia learning, further subdividing SRL into cognition, metacognition, and motivation. This framework has proven particularly useful in guiding the analysis of trace data gathered in hypermedia environments to understand learners' SRL processes (Bannert et al., 2014; Fan et al., 2022a; Srivastava et al., 2022; Lim et al., 2023). Consequently, cognitive and metacognitive processes of SRL can be operationalized as patterns and sequences of learning actions, encompassing activities such as orientation—collecting information about the learning task to implement SRL strategies—and evaluation—monitoring learning progress throughout the learning process (Siadaty et al., 2016b; Saint et al., 2020; Fan et al., 2022a; Srivastava et al., 2022).

The beneficial impact of SRL on academic performance has been frequently highlighted in the literature (Greene and Azevedo, 2007, 2009; Broadbent and Poon, 2015; Broadbent, 2017; Maldonado-Mahauad et al., 2018). Moreover, compared to other intrinsic factors such as self-efficacy and motivation, the ability to use effective SRL processes in learning are considered to be more predictive of successful academic performance (Pintrich and De Groot, 1990). However, literature has shown that many students find it difficult to use SRL processes without guidance (Greene and Land, 2000; Bannert, 2009; Jovanović et al., 2017; Guo, 2022). Thus, there is a need to aid learners in their development of SRL.

As suggested by Bannert and Reimann (2012), effective SRL instruction should encompass several facets: (1) integration—contextually integrating with the specific learning domain; (2) explanation – elucidating how the suggested SRL processes can be effectively applied; and 3) training—providing ample training such that learners' can effectively use SRL processes. Scaffolding, defined as structured guidance for acquiring skills within a specific learning context until they can perform independently (Pea, 2004), may offer a viable solution to these instructional necessities.

### 1.2. Adaptive scaffolding

Research suggests that SRL scaffolding is positively associated with improvements in academic performance and learning processes. Specifically, SRL scaffolding has been examined from various perspectives, such as the persistence of scaffolding effects (Bannert et al., 2015; Sonnenberg and Bannert, 2019), effectiveness of technological scaffolds (Milikić et al., 2018; Lahza et al., 2022), the utility of scaffold training (Bannert, 2009), impact on group activities and group performance (Molenaar et al., 2011), influence of demographic factors (Pieger and Bannert, 2018), and association with different goal orientations (Duffy and Azevedo, 2015). Moreover, the effects of scaffolding have been examined in diverse contexts, including educational settings (Azevedo et al., 2004; Bannert, 2009; Sonnenberg and Bannert, 2016) and workplaces (Siadaty et al., 2016a,b). However, a critical characteristic of the previous studies is that the scaffolding was primarily *fixed*—i.e., the content of the scaffolding was the same for each student, and the design of the content was largely informed by the findings from the existing literature (Bannert, 2009; Bannert et al., 2015; Pieger and Bannert, 2018; Guo, 2022).

Wong et al. (2021), for example, divided learners into three groups — fixed question prompt, fixed recommendation prompt, and no prompt—and investigated the effect of each compared to a control condition. Their results suggested that neither type of fixed scaffolding significantly affected SRL processes, such as time-management, self-reflection, planning, and self-monitoring. The authors concluded that scaffolding designed to flexibly target specific SRL processes could be more effective. Similarly, a recent systematic literature review by Guo (2022) found that the adaptivity of scaffolding is a strong moderator of the relationship between SRL processes and learning. Thus, *adaptive scaffolding*, which responds to actions of individual learners and targets their specific deficiencies, may be more effective in supporting learners' SRL processes (Guo, 2022).

Although some previous studies have examined the effects of adaptive scaffolding on SRL processes, this study has several limitations. First, early studies implemented adaptive scaffolding that was only partially automated, limited their use in larger-scale learning contexts, confounding the results. For example, Azevedo et al. (2004) investigated differences in learners' SRL processes among three scaffolding conditions (adaptive scaffolding, fixed scaffolding, and no scaffolding) and found that learners who received adaptive scaffolding from human tutors more frequently regulated their learning by activating prior knowledge, utilized more diverse learning strategies, and engaged in more help-seeking behaviors. However, the researchers caveated their findings by suggesting that their positive findings could have been due to the presence of the human tutor.

More recently, the adoption of adaptive scaffolding has gathered momentum through the implementation of automated rule-based algorithms. For example, Duffy and Azevedo (2015) implemented rule-based adaptive scaffolding and found that learners who received this kind of scaffolding used more SRL strategies and spent more time viewing the learning material. Munshi et al. (2023) also designed an automated rule-based adaptive scaffolding system that tailored SRL suggestions to learners. Specifically, the adaptivity is based on learners' real-time learning behaviors, and learners were offered a procedure or a piece of knowledge that they struggled to properly apply. For instance, when a learner employed an ineffective strategy during a task, such as adding erroneous elements during knowledge construction, immediate scaffolding guided them toward self-assessing their understanding.

While these studies are a step forward in investigating the effectiveness of automated adaptive scaffolding, they were limited because they were only compared to control conditions in which no scaffolding was provided. To more completely understand the utility of automated adaptive scaffolding, it needs to be compared to both a control condition with no scaffolding *and* a condition in which fixed scaffolding is provided.

### 1.3. Segmentation analysis

In addition to the limitations described above, prior studies have focused on the effects of scaffolding using data aggregated over the entire learning task (Sitzmann et al., 2009; Azevedo et al., 2011; Molenaar, 2014; Duffy and Azevedo, 2015; Sonnenberg and Bannert, 2016; Srivastava et al., 2022). For instance, Sitzmann et al. (2009) assessed the effectiveness of adaptive scaffolding by measuring the activation of SRL processes through a questionnaire provided at the end of a learning task. In another study, Duffy and Azevedo (2015) extracted log file data and measured learners' SRL processes based on the overall frequency of several learning behaviors. In contexts where multiple scaffolds are provided throughout the learning task, this practice limits our understanding of how each specific scaffold is associated with SRL processes. In other words, this approach results in an “averaging out” of the detailed effects of scaffolding.

To address the above limitation, some have suggested segmenting the learning task for analysis to provide better insights into the effects of the individual scaffolds (Molenaar, 2014; Knight et al., 2017; Saint et al., 2022). In the context of implementing SRL scaffolds, segmentation involves dividing the overall learning task into multiple segments based on appropriately defined time windows (e.g., between scaffolding events) and then examining the SRL processes within each segment (Knight et al., 2017; Fincham et al., 2018; Saint et al., 2022). Once the overall learning task is divided into segments, researchers can identify the immediate or lagged changes in SRL processes after each scaffold to evaluate the association between specific scaffolds and SRL processes. This approach can increase our understanding of the effect of each individual scaffold (Saint et al., 2022).

Despite these potential benefits, few studies have implemented such a segmentation approach. Among them, two major limitations still remain. First, previous studies have largely focused on changes in general cognitive behaviors, overlooking changes in metacognitive or SRL processes. For example, Munshi et al. (2023) implemented six scaffolds throughout the learning task and evaluated the behavioral changes immediately after each scaffold. However, this study only examined changes in learning behaviors prompted by each scaffold (e.g., if the scaffold suggests that learners take a quiz to assess their understanding, how many learners took the quiz?) and did not address changes in SRL processes (e.g., if the scaffold suggests that learners should monitor their learning processes, to what extent do they adopt a more monitoring-oriented learning process). Second, segmentation analysis has mostly been conducted over longer learning periods – either segmenting a whole learning semester into different weeks (Mahzoon et al., 2018) or a whole week into different days (Dorodchi et al., 2018), while few studies have implemented a lower-level segmentation analysis of a single learning task. Conducting such studies at the segment-level should provide a more detailed information about the effect of specific scaffolds on SRL behaviors in addition to the general effects of scaffolding.

### 1.4. Research questions

In this study, we sought to address the limitations of a prior study that investigated the effect of adaptive scaffolding on SRL processes. To do so, we collected learner interactions (i.e., trace data) with an online environment and compared the SRL processes of learners in three conditions—an adaptive scaffolding condition (AS), a fixed scaffolding condition (FS), and a control condition (CN) in which no scaffolding was provided. This analysis was conducted at two levels: (i) the overall task level and (ii) the task segment level, where segments were defined according to the timing of the scaffolding. Our study was guided by the following research questions:

1. How is adaptive scaffolding, compared to fixed scaffolding and no scaffolding, associated with SRL processes when analyzed at the *task* level?
2. How is adaptive scaffolding, compared to fixed scaffolding and no scaffolding, associated with SRL processes when analyzed at the *task segment* level?

At the task level, we hypothesize that learners who receive adaptive scaffolding will be more likely to engage in high-cognitive and metacognitive SRL processes [as defined in Bannert's SRL model (Bannert, 2007)] compared to those receiving fixed or no scaffolding. Our hypothesis is grounded in existing literature (Sonnenberg and Bannert, 2015; Siadaty et al., 2016a; Wong et al., 2021). For example, Siadaty et al. (2016a) identified that those who received technological scaffolding exhibited more micro-level SRL processes within the forethought or preparatory phase of SRL. At the task segment level, we hypothesize that learners who receive adaptive scaffolding will tend to comply with the scaffolding recommendations more so than those in the other conditions. In other words, when examining differences at the task segment levels, which are defined by time periods between scaffolds, we expect the SRL processes of students in the adaptive condition to align more with the most recent scaffold they received because it was tailored to their prior behaviors. These hypotheses are grounded in the adaptive nature of scaffolding that tailors its assistance to address the unique needs and learning gaps of each individual learner (Duffy and Azevedo, 2015; Guo, 2022; Lim et al., 2023; Munshi et al., 2023).

Previous research has highlighted the necessity of recognizing the relationships between the SRL process that learners use instead of viewing these processes in isolation (Saint et al., 2022). This conceptual shift is vital because a learner might appear to be engaged in, for example, re-reading the text while also checking task instructions to understand its key aspects. Therefore, taking the co-occurrence of multiple SRL processes into consideration is critical. Moreover, previous studies have acknowledged that SRL processes are context-sensitive and sequence-specific—that is, any given SRL process can precede or follow other SRL processes, and different orders imply different meanings (Fan et al., 2023). To account for the connected and sequential nature of SRL processes, we used the network analytic technique, *ordered network analysis* (ONA) (Tan et al., 2023). This technique, as well as the details of our experimental design and data, is described in the sections below.

## 2. Materials and methods

### 2.1. Research context and design

#### 2.1.1. Participants

We conducted this study with participants from a graduate level academic writing course at a large university in China. Participants were non-native English speakers. The expected learning outcome of the course was to improve the academic writing skills of first-year graduate students, for whom English was not their first language. As part of the course, participants were tasked with completing a writing assignment on a Moodle-based learning platform that integrated instrumentation tools and learning analytics-based scaffolding (a detailed description of the learning platform is summarized in Section 2.1.2). This study received approval from the ethics committee prior to the commencement of data collection.

Participants for this study were recruited from two separate offerings of the same course (the first round in November 2021 and the second round in April 2022). Consequently, the course design, task design, and learning context covered in both rounds of data collection were identical. The participants hailed from various disciplinary backgrounds and did not receive monetary incentives for their participation in the study. A total of 437 students (137 from round 1 and 300 from round 2) participated in two rounds of data collection, which resulted in a total of 161 valid participants whose data were complete and usable. The data of 276 participants were excluded because they i) did not consent to their data to be analyzed; ii) technical errors (e.g., incomplete data records or scaffolding not successful triggered); or iii) did not submit a complete writing product. A summary of participant numbers is presented in Table 1. Overall, the participants for each group were randomly assigned and similarly distributed across the study conditions: 53 learners in the control (CN) group (32 from round 1 and 21 from round 2), 57 learners in the fixed scaffolding (FS) group (28 from round 1 and 29 from round 2), and 51 learners in the adaptive scaffolding (AS) group (22 from round 1 and 29 from round 2). The sample consisted of 55 percent female and 45 percent male university students, with minority ethnic groups comprising 12 percent of the population. Their academic majors were diverse, spanning physics, engineering, ecology, and computer science, among others.

**Table 1:** Summary of participant information across two rounds of data collection.

**Round**	**Group**	**Participants**	**Excluded**	**Valid participants**
1 - 2022 Apr	Control	32	0	32
1 - 2022 Apr	Fixed	60	32	28
1 - 2022 Apr	Adaptive	45	23	22
2 - 2021 Nov	Control	91	70	21
2 - 2021 Nov	Fixed	103	74	29
2 - 2021 Nov	Adaptive	101	72	29
2 - 2021 Nov	Admin and NA	5	5	0
Total		437	276	161

#### 2.1.2. Learning platform and task design

**Figure 1:**
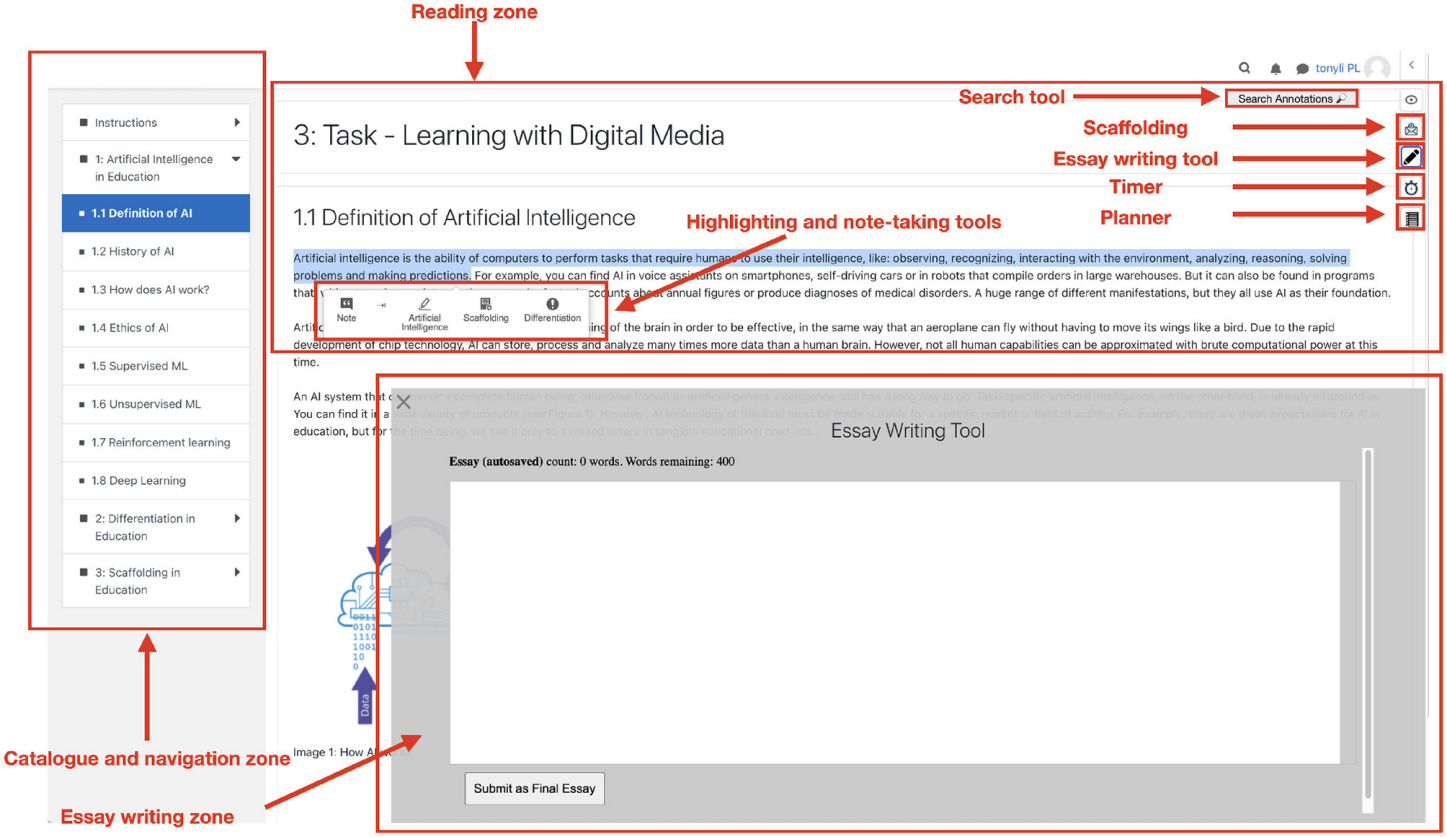
Snapshot of the learning environment.

The learning platform used in this study was an extended version of the Moodle learning environment, where participants were asked to complete their writing assignment. As depicted in Figure 1, the platform interface consisted of several main functional zones, including the catalog and navigation zone for learners to navigate and access reading materials, the reading zone for displaying content and enabling learners to use annotation tools for note-taking or highlighting, and the essay writing zone for learners to compose their essays. The platform also incorporated various instrumentation tools, such as a search tool, timer, planner, and scaffolding tool. Such instrumentation tools have been shown useful for capturing trace data and measuring learners' SRL processes (van der Graaf et al., 2021). Lastly, scaffolding was provided to learners via a pop-up window, prompting them to regulate their SRL processes. Detailed explanations regarding the types of scaffolding deployed on the platform are presented in the Section 2.1.3.

Participants in this study were asked to complete four activities related the topic of AI and education: (i) a pre-task activity that consisted of a pre-survey, a pre-knowledge test including 10 multiple choice questions about AI and education and a consent form for participation; (ii) a training activity where participants were instructed on how to use and interact with those embedded instrumentation tools (e.g., how to create annotations and tags); (iii) a two-hour main task activity that involved reading and writing, i.e., a 300–400 word essay on AI and education; and (iv) a post-task activity consisting of a post-task knowledge test including 10 multiple choice questions about AI and education, a transfer test (10 multiple choice questions about the application of AI in medicine), and a post-task survey. The training on how to interact with scaffolds in the training activity was deemed crucial as previous studies have shown that the effectiveness of scaffolds on academic outcomes improves when learners receive prior training (Bannert, 2009). For the main writing task, participants were provided with reading materials covering three topics—AI in education, differentiation in education, and scaffolding in education. Based on these materials, participants were asked to compose the essay. The main task was set with a time limit of 120 minute, and the average time spent on the main task was 113 minute. Given that the participants were non-native English speakers, and the task was conducted in English, this imposed an inherent time pressure. This pressure was further amplified by the considerable volume of text contained within the reading materials. We purposefully designed the task this way to encourage participants to adopt a selective reading approach, guided by the task instruction and/or rubric.

#### 2.1.3. Scaffolding design

**Figure 2:**
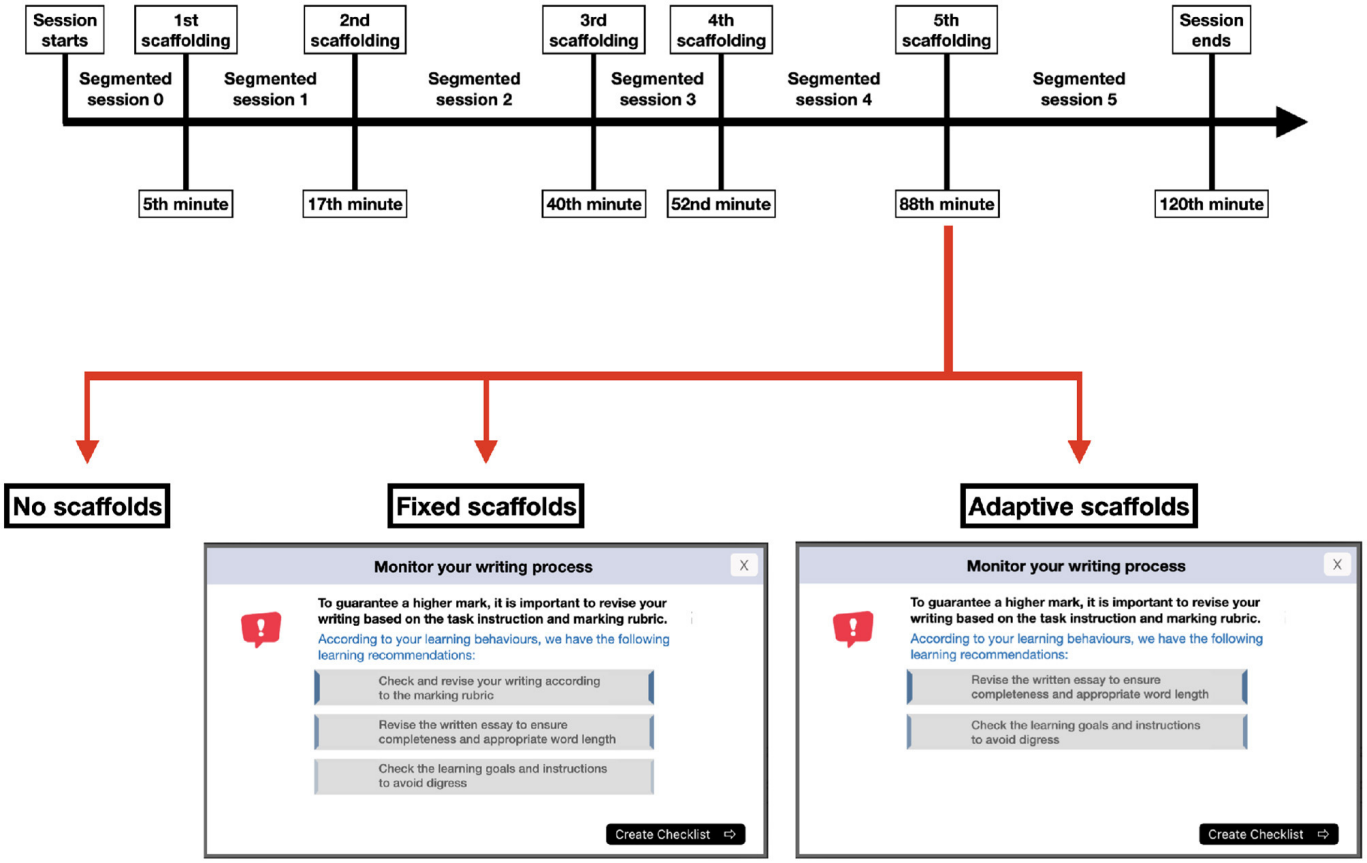
Segmentation and scaffolding design example.

Participants in this study were allocated to one of three study conditions: the CN, FS, and AS groups. The CN participants received no scaffolding. For the FS group, scaffolds were not differentiated among different participants—everyone received the same scaffolds that were designed according to the participants' general learning needs as referenced from the relevant literature and lab studies (van der Graaf et al., 2022). Lastly, the AS group participants received personally tailored scaffolding, the adaptivity of which was determined by an algorithm implementing a rule-based approach. This algorithm included relevant suggestions in the scaffolds based on real-time analysis of SRL processes derived from the three types of trace data (see Section 2.2). The scaffolding was delivered via pop-up windows, and the scaffolding content in the AS group was adaptively adjusted according to observed SRL processes in trace data. Figure 2 illustrates an example of the differences between fixed and adaptive scaffolding pop-up windows. Fixed scaffolds were presented with all learning suggestions (e.g., check and revise your writing according to the marking rubric) that were posited to be useful to learners regardless of their SRL processes. In contrast, if the real-time analysis based on trace data revealed that learners had performed certain SRL processes (e.g.,check and revise your writing according to the marking rubric), the adaptive scaffolds would hide relevant prompts and would only suggest SRL processes that had not been observed in trace data. In cases where learners performed all three suggested SRL processes before the triggering time of the scaffolds, the scaffold windows were hidden.

For FS and AS, five scaffolds were embedded within the main task activity (Table 2). The timing for each scaffold was fixed at 5th, 17th, 40th, 52nd, and 88th minute, which also guided the timing of our segmentation for data analysis (Figure 2). The timing and content design of the scaffolds were informed both theoretically, by aligning with the cycle of SRL (Winne, 1997), and empirically, by incorporating findings from previous studies (Lim et al., 2021; Srivastava et al., 2022). For instance, high-performing learners were shown to employ more metacognitive strategies, such as monitoring their rereading processes and selectively reading, compared to less successful learners (Lim et al., 2021). In response, the third scaffold was designed to prompt learners to monitor their reading process. Similarly, a prior lab study identified a positive association between essay revision and academic performance, as measured by the essay score (Raković et al., 2022). Accordingly, the last scaffold was created to prompt learners to review their written work before submission. Each scaffold had a distinct theme (i.e., purpose), including understanding the task (1st), starting to read (2nd), monitoring the reading process (3rd), starting to write (4th), and monitoring the writing process (5th). Within each scaffold, based on its main theme, three suggestions were included (Table 2).

**Table 2:** Content of the scaffolding.

**Scaffold number and timing**	**Theme**	**Main message**	**Learning prompts**
First Scaffolding at the 5th minute	Understand the task	It is important to understand the learning content and requirement. According to your learning behaviors, we have the following learning recommendations.	(a) Use the navigation tool to generate an overview impression of the task; (b) Read the marking rubric carefully; (c) Ensure a clear understanding of the learning goals and task instructions.
Second Scaffolding at the 17th minute	Start reading	It is necessary to read information on different topics in the material efficiently and with high quality. According to your learning behaviors, we have the following learning recommendations.	(a) Use annotation tool to take notes on key information; (2) Use navigation tool to guide your reading; (c) Use timer to monitor your reading progress.
Third Scaffolding at the 40th minute	Monitor reading process	It is important to read selectively and focus on task-related pages and to remind yourself with the reading-and-writing relationship. According to your learning behaviors, we have the following learning recommendations.	(a) Review annotations to monitor what have already been learned; (b) Ensure you are reading relevant pages by reviewing the learning goals and task requirements; (c) To read selectively as informed by your writing progress and your overall conception on the task.
Fourth Scaffolding at the 52nd minute	Start writing	The key to the success of this assignment is to start your writing early and to write in high quality. According to your learning behaviors, we have the following learning recommendations.	(a) Use the timer to monitor your writing progress; (b) Review the marking rubric page; (c) Paraphrase the main arguments that you have read and write in your own words.
Fifth Scaffolding at the 88th minute	Monitor writing process	To guarantee a higher mark, it is important to revise your writing based on the task instruction and marking rubric. According to your learning behaviors, we have the following learning recommendations.	(a) Check and revise your writing according to the marking rubric; (b) Revise the written essay to ensure completeness and appropriate word length; (c) Check the learning goals and instructions to avoid digress.

### 2.2. Data collection and analysis

#### 2.2.1. Data collection

Data were collected and processed according to the trace-based SRL measurement protocol (see the Electronic Appendix at this link, containing an Action Library and a Process Library, which make up the trace parser) to generate SRL process data for each participant (Siadaty et al., 2016b; Saint et al., 2020; Fan et al., 2021b, 2022a,b). This study collected three types of trace data: (1) time-stamped navigational logs (i.e., clickstreams), (2) mouse traces incorporating mouse movements and scrolls, and 3) keyboard strokes. The selection of these data types was based on Winne and Hadwin's model of SRL (Winne and Hadwin, 1998), which posits that learning conditions, learners' operations on information, and the standards they employ for self-evaluation are adjustable and typically vary over time (Winne, 2017). Therefore, considering SRL as a dynamic process, it is crucial to collect time-stamped learning trace data to monitor alterations in learning behavior. Once collected, the data were processed following the trace parser, grounded theoretically in Bannert's self-regulated hypermedia learning framework (Bannert, 2007) and adopted in preceding studies (Fan et al., 2022a; Srivastava et al., 2022). The trace parser facilitated the processing of learning trace data through the action and process libraries, mapping trace data onto SRL processes. The validity of collecting trace data and processing via the trace parser has been affirmed in previous studies using think aloud data (Fan et al., 2022b). Because not all AS learners were presented with every scaffold, for each segment, the data from learners who had not received the corresponding scaffold were excluded. For instance, if a learner has shown all the anticipated SRL processes for the upcoming fifth scaffold, the scaffolding window would be concealed from that learner. Consequently, that learner's trace data in the fifth segment would not be included in our analysis. Table 3 summarized the number of scaffolds that have been triggered for AS learners in each segment.

**Table 3:** Count of AS learners receiving each scaffold across two rounds of data collection.

**Rounds**	**Scaffold 1**	**Scaffold 2**	**Scaffold 3**	**Scaffold 4**	**Scaffold 5**
Round 1	22	20	13	7	3
Round 2	29	29	25	16	6

For CN learners, they did not receive any scaffolds. For FS learners, they received all five scaffolds.

#### 2.2.2. Data analysis

After the trace data were coded for SRL processes via the protocol, we analyzed the data using ONA to compare the SRL processes between AS and CN groups, as well as between AS and FS groups. A detailed technical description of ONA is beyond the scope of this study. For more information, see the study by Tan et al. (2023), which describes ONA, as well as the study by Shaffer et al. (2016), which describes epistemic network analysis (ENA), the widely used learning analytic technique on which ONA is based.

Briefly, ONA builds on ENA to measure and visualize the frequency of transitions between coded events in the data. Transitions are represented as points in a low-dimensional space (i.e., embeddings) and as network diagrams, whose nodes correspond to the codes and whose edges correspond to the relative frequency of transitions between codes. We chose ONA over other common approaches such as process mining to analyze SRL processes because previous studies had demonstrated data analytic and visual advantages of ONA. For example, Fan et al. (2023) applied ONA in analyzing SRL tactics used by MOOC learners and found that ONA revealed insights about the frequency, continuity, sequentiality, and role of different learning actions in learning tactics that other techniques such as process mining failed to fully represent.

We conducted the analysis using the ONA package for the programming language R (Marquart et al., 2023). The codes described in Table 4 are represented as nodes in the resulted ONA networks. To measure transitions between codes, ONA constructs ordered networks to represent the directed and weighted co-occurrence among coded events within pre-defined segments of data. Any transitions that occur within these segments are counted and contribute to the weight of the resulting network edges.

**Table 4:** SRL processes that were measured in the current study were based on the coding scheme proposed by Bannert et al. (2014).

**Main category**	**Sub-category**	**Definitions**
**Metacognition**	Orientation	Orientation on the learning-related activities, on prior knowledge, on the task and feeling about the task. For example, after reading the general instruction page, learners read through the catalog (i.e., the navigation zone) to get a overview of what topics they need to learn and then read some pages.
	Planning	Planning of the reading and writing process by arranging activities and determining strategies—for example, using the planner tool to make a plan.
	Evaluation	Checking of content-wise correctness (e.g., the essay content) of learning activities—for example, learners check instruction/rubric when they run into read some irrelevant pages then move on to read some relevant pages.
	Monitoring	Monitoring and checking the reading and writing progress—for example, checking the timer or planer tool, or searching and reading annotations.
**Low cognition**	First-reading	Reading information or figures for the first time—for example, reading new content.
	Re-reading^*^	Rereading of information in the text of figures—for example, re-reading or reviewing content that has been read.
**High cognition**	Elaboration/ organization	Elaborate and organize by connecting content-related comments and concepts during reading or writing. For example, using annotation tools to label and edit annotations, or writing essay.

These processes are measured using the multichannel data (navigation logs, mouse movements, and keystrokes) following the protocol proposed by Siadaty et al. (2016b), Saint et al. (2020).

* Re-reading is operationalized when a learner spent more than 6 seconds on a page.

To address RQ1, we conducted ONA analysis on the task-level of SRL processes, meaning all transitions within the task were counted. To address RQ2, only transitions that occurred within the same task segment were counted. Although the length of segmented sessions that are involved in the analysis varied from 12 to 36 minute, learners were working on an independent writing task in a structured learning environment without external interruptions. As explained in a previous section, it was plausible for AS learners to not receive one or more scaffolds if they had already demonstrated all expected SRL processes before the corresponding scaffold was triggered. Consequently, in cases where AS learners did not receive a particular scaffold, their corresponding trace data for that segment was excluded from the analysis. For example, if an AS learner did not receive the third scaffold, their trace data from the third segment was removed.

In ONA networks, the directed transitions between codes are represented by tapered edges. The chevron on each edge indicates the direction in which the transition occurred most frequently. For example, a chevron on the edge for first-reading/monitoring pointed toward monitoring indicates that more individuals transitioned from first-reading to monitoring rather than the other way around. Thicker and more saturated edges indicate that the connection occurred more frequently. The size of a given node in the network is proportional to the number of occurrences of that code in the data. The larger the node is, the more times that code followed prior events. The colored circle inside a node represent self-transitions—i.e., repeating the same SRL process. A larger circle means more self-transitions.

The node placement in ONA is the same for each unit of analysis—here, individual learners—facilitating comparisons between networks. Networks can be compared by subtracting their edge weights to find the edges that are stronger in one network vs the other. Additionally, it is possible to average individual networks—by averaging their edge weights—to compare the overall transition patterns between subgroups in the data. In this study, we averaged the networks of individual learners in each experimental condition for comparison. The network edges shown in this study were scaled by multiplying the same constant with each network. This process retained the relative differences among connections and plots while making the network graphs more readable.

Finally, because ONA also creates low-dimensional embeddings for each network using dimensional reduction via singular value decomposition, statistical comparisons can be made between groups of networks. In this study, we compared the average embeddings for each condition using Mann-Whitney tests. These tests indicate whether the pattern of transitions each condition made were significantly different. These tests were conducting using the positions of the embeddings on the first and second dimensions of the embedding space. These dimensions account for the most variance among the units of analysis, and they can be interpreted using the positions of the network nodes in the space. Nodes—and the transitions they represent—that are on the extremes of the dimensions are the most influential at distinguishing between units of analysis.

## 3. Result

To address our research questions, we created ONA network subtractions that visually compared the mean network of the AS condition to the mean networks of the FS and CN conditions at the task and segment levels. We conducted Mann-Whitney U tests between the mean networks using their embedding values on the first and second dimensions of the ONA space. The statistical analyses carried out in this study, encompassing mean differences, *p*-values, effect sizes, and power calculations, are presented in Table 5. All tests were conducted using a Bonferroni correction to control for family-wise error, where each family consisted of the given level (e.g., task-level or segment 4) and four tests. Power analyses were conducted using the statistical software GPower 3.1.

**Table 5:** Statistical result for both task-level and segment-level models.

**Level**	**Comparison**	**Dimension**	**Estimate**	***p*-value**	**Effect size (*d*)**	**Power (1 − β)**
Task-level	AS vs. CN	1	0.015	0.7341	0.034	0.02
Task-level	AS vs. CN	2	0.112	0.000^*^	0.312	0.82
Task-level	AS vs. FS	1	0.025	0.5379	0.056	0.03
Task-level	AS vs. FS	2	0.125	0.000^*^	0.348	0.91
Segment 4	AS vs. CN	1	0.082	0.1416	0.318	0.1
Segment 4	AS vs. CN	2	0.083	0.2054	0.30	0.09
Segment 4	AS vs. FS	1	0.096	0.1420	0.372	0.07
Segment 4	AS vs. FS	2	0.004	0.9389	0.014	0.01
Segment 5	AS vs. CN	1	0.051	0.1674	0.378	0.07
Segment 5	AS vs. CN	2	0.059	0.5828	0.324	0.05
Segment 5	AS vs. FS	1	0.057	0.3024	0.422	0.08
Segment 5	AS vs. FS	2	0.070	0.6728	0.385	0.07

* Indicates a significant result at the α = 0.0125 level.

### 3.1. RQ1: Effectiveness of scaffolding at the task level

#### 3.1.1. Comparison between AS and CN groups

**Figure 3:**
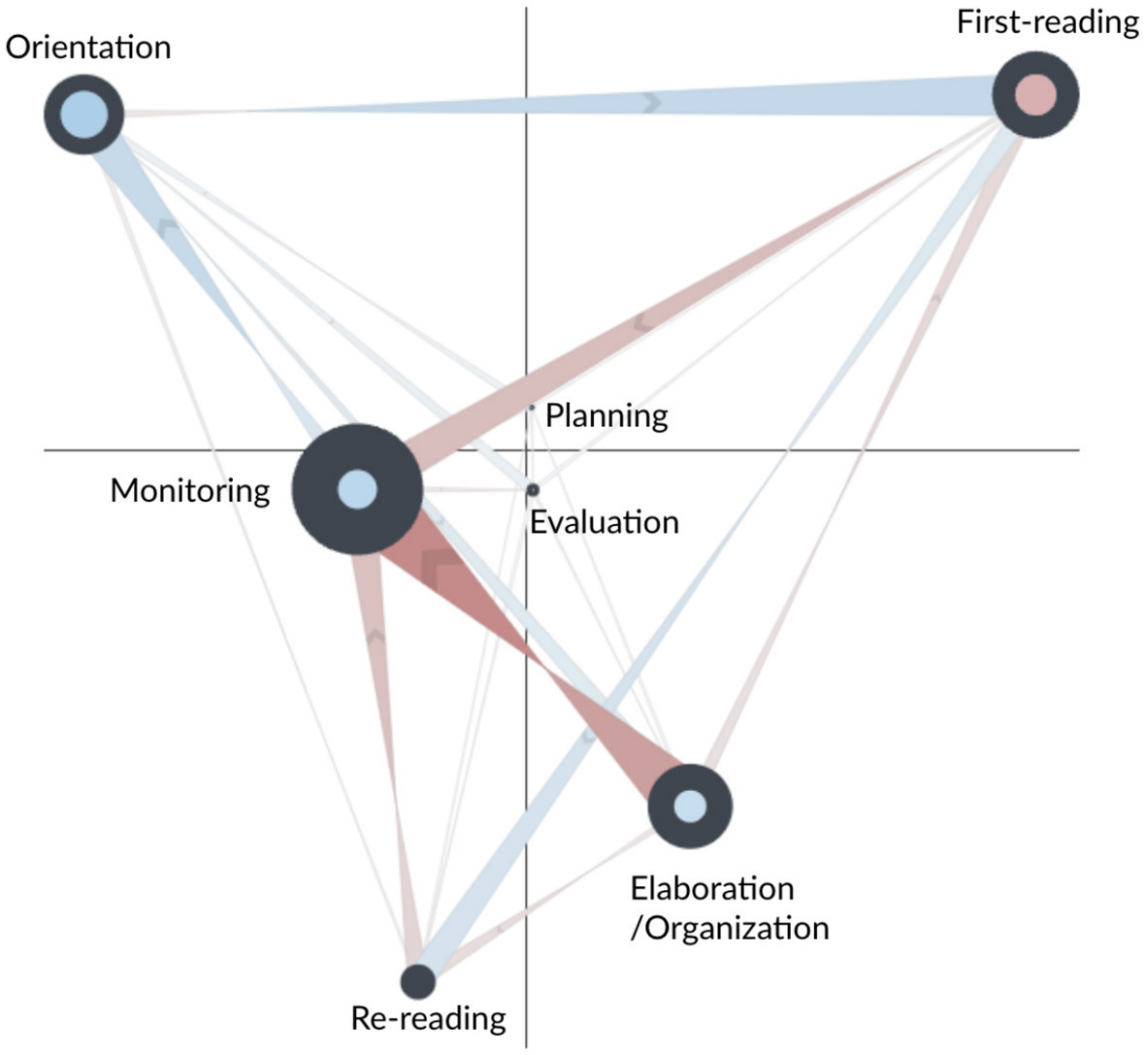
Subtracted ONA network of SRL process between the control (CN, in red) and adaptive scaffolding (AS, in blue) groups for the whole main task.

The network subtraction for the AS and CN learners is shown in Figure 3. Blue edges represent more frequent transitions for the AS learners, while red edges indicate more frequent transitions for the CN learners. The network subtraction shows that CN learners made more frequent self-transitions to first-reading, suggesting a sequential reading approach during some parts of the task. They also made more frequent transitions from first-reading to monitoring and from re-reading to monitoring, suggesting that they were more engaged in monitoring their reading processes throughout the task, for instance, by checking the remaining time. Similarly, compared to AS learners, CN learners made more frequent transitions between monitoring and elaboration/organization. As elaboration/organization involves essay writing as recorded from keystrokes, this strong transition suggests a recurrent cycle of writing, time-checking, and returning to writing processes.

The figure shows that AS learners made more frequent transitions to orientation (self), from monitoring to orientation, and from orientation to first-reading. This suggests that they tended to use the catalog and navigation window to guide their reading and writing process, and they regularly incorporated task instructions/rubrics in their reading process. More frequent self-transitions to monitoring highlight AS learners' deeper engagement with actions such as navigating to specific pages, referring to previously created notes, searching through annotations, and checking the timer. This pattern points to a more layered, detailed approach to self-regulation among AS learners. In contrast, CN learners mainly exhibited transitions to the monitoring node but not self-transitions within it. This suggests that CN learners also engaged in monitoring their learning progress during their reading and writing processes—but this monitoring tended to occur on an as-needed basis rather than being a consistent, deeply engaged activity as seen in the AS learners. AS learners also made more frequent self-transitions to elaboration/organization, suggesting more involvement in writing and note-taking processes. Moreover, stronger transitions from first-reading to re-reading suggest that they tended not to read sequentially. Instead, they tended to revisit previously read information, indicating a deeper, more thoughtful engagement with the material. In contrast, the CN learners predominantly followed a linear, page-by-page reading strategy, suggesting less thorough engagement with the material.

The statistical test (second row of Table 5) indicates that the AS and CN learners differed significantly in their processes along the second dimension of the ONA space. CN learners tended to make more transitions that involved elaboration/organization, monitoring, and re-reading, whereas AS learners tended to make more transitions that involved orientation and first-reading. Taken together, the results suggest that CN learners typically engaged in a sequential and reactive learning approach, often monitoring their progress during reading, re-reading, and writing tasks. Conversely, AS learners demonstrated a deeper, more reflective learning process, regularly integrating task instructions/rubrics into their reading, revisiting previously read information, and partaking in a broad range of monitoring processes.

#### 3.1.2. Comparison between AS and FS groups

**Figure 4:**
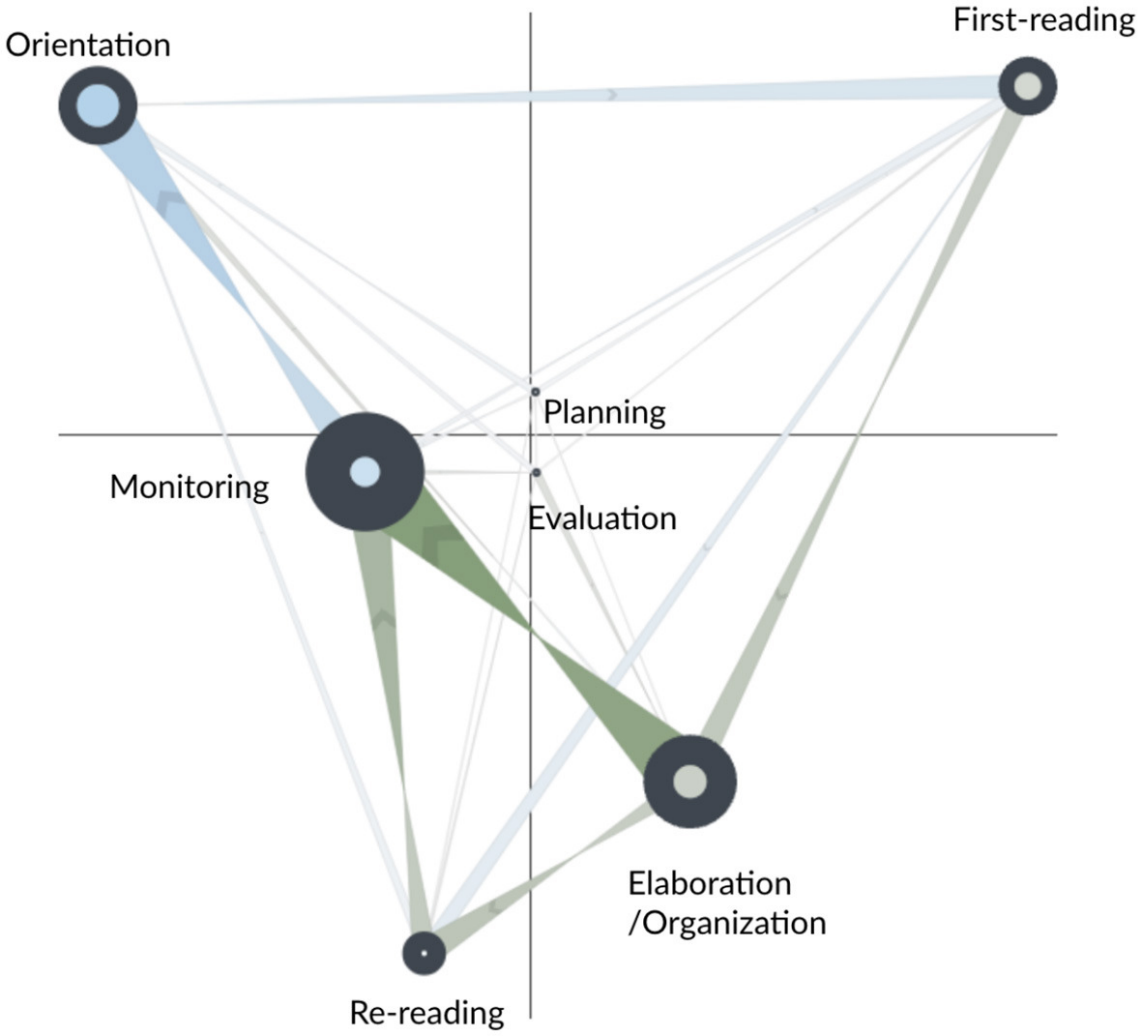
Subtracted ONA network of SRL process between the fixed scaffolding (FS, in green) and adaptive scaffolding (AS, in blue) groups for the whole main task.

The network subtraction for the AS and FS learners is shown in Figure 4. Blue edges represent more frequent transitions for the AS learners; green edges indicate more frequent transitions for the FS learners. The figure suggests that the FS learners had similar transition patterns to the CN group and that the differences between the AS and FS groups are similar to the differences described above. The one exception is that FS learners did not make more frequent transitions from first reading to monitoring—the thin and faint edge for this transition indicates that AS and FS learners had similar amounts for this transition.

The statistical test (fourth row of Table 5) indicates that the two groups were significantly different along the second dimension of the space—FS learners tended to make more transitions that involved elaboration/organization, monitoring, and re-reading, while AS learners tended to make more transitions that involved orientation and first-reading. Similar to above findings, the results suggest that FS learners typically engaged in a sequential and reactive learning approach—monitoring their progress after re-reading and writing. AS learners, on the contrary, demonstrated a deeper, more reflective learning process, regularly integrating task instructions/rubrics into their reading, revisiting previously read information, and partaking in a broad range of monitoring processes.

### 3.2. RQ2: Effectiveness of scaffolding at the segment level

To further investigate how each scaffold was associated with learners' SRL processes, we segmented the data based on the timing of each scaffold. This resulted in five segments, five corresponding network subtractions between the AS and CN learners, and five corresponding network subtractions between the AS and FS learners. All visualizations are included in the Appendix at this link. To address RQ2, our analysis focuses on the segments occurring after the triggering of the fourth and fifth scaffolds. This selection assumes that later segments are likely to include more diverse SRL processes. As shown in Table 5, no comparisons between the scaffolding groups in these segments were statistically significant. However, the small-medium effect sizes for these results and low statistical power suggest that, if we had more data, these results would be significant. Thus, we still describe the network subtractions as they suggest differences that may become more salient in future studies.

#### 3.2.1. Comparing CN and AS groups post the fourth scaffold

**Figure 5:**
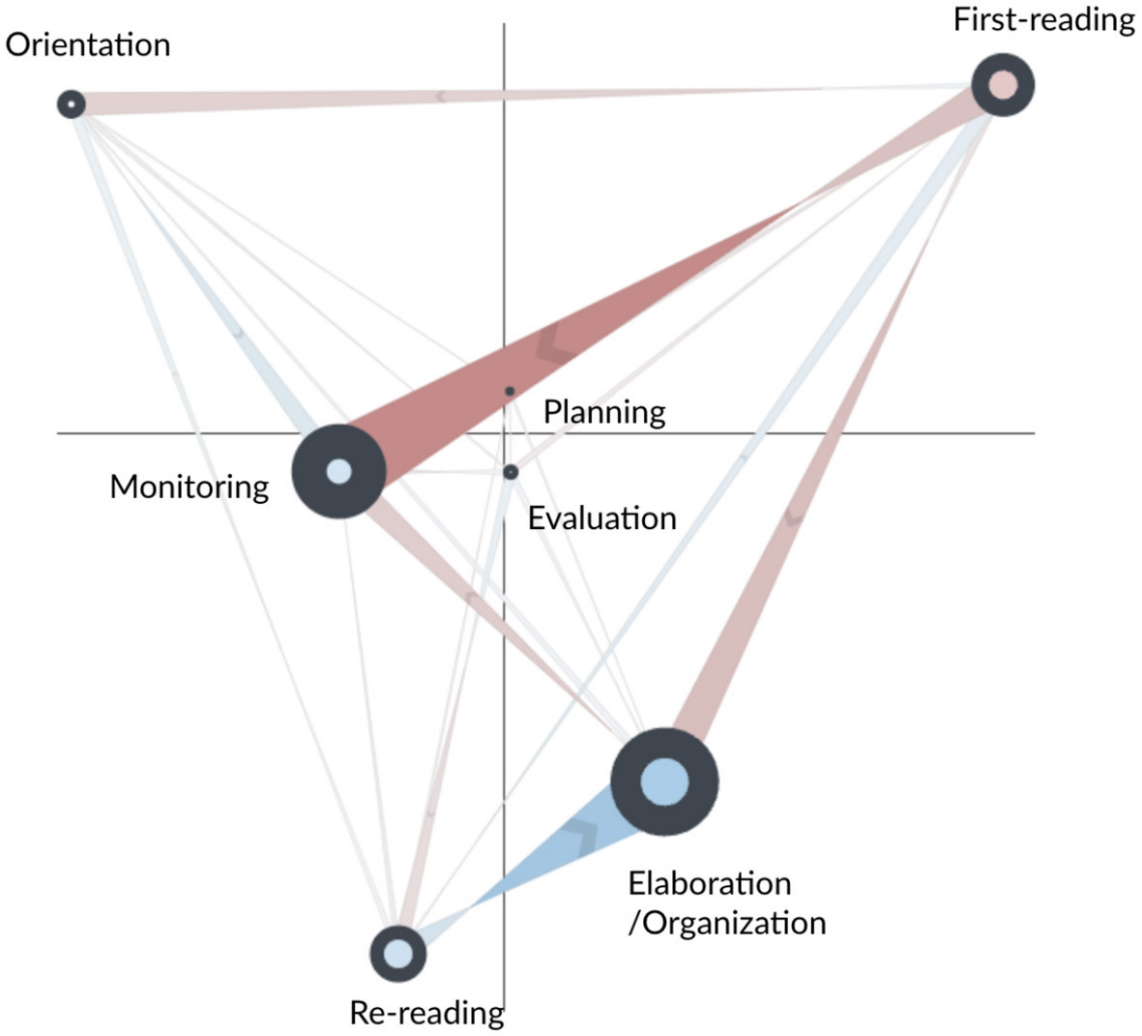
Subtracted ONA network of the SRL process between the control (CN, in red) and adaptive scaffolding (AS, in blue) groups during the fourth segment of the study task.

Figure 5 shows the network subtraction for the CN and AS learners at the end of the fourth segment (CN in red; AS in blue). For AS learners, this is the learning stage where they have just received the fourth scaffold, which depending on their prior actions, prompted them to commence writing and do so strategically by employing a range of self-regulated learning techniques.

CN learners made stronger self-transitions to first-reading and stronger transition from first-reading to monitoring. This suggests that, at this learning stage, there were still heavily engaged in continuous, page-by-page reading while frequently monitoring their reading progress by, for example, checking the remaining time. Furthermore, CN learners made stronger transitions from first-reading to elaboration/organization, indicating that they were creating notes and/or gradually starting writing based on the information they read page-by-page.

AS learners made stronger self-transitions to re-reading and elaboration/organization and stronger transitions from re-reading to elaboration/organization. These transitions suggest that upon receiving the fourth scaffold, AS learners initiated their essay writing process by referring back to previously read pages. This diligent reviewing, understanding, and organizing of their essay content aligned with the recommendations provided in the fourth scaffold, which encouraged learners to' paraphrase the main arguments that you've read and write in your own words'.

#### 3.2.2. Comparing CN and AS groups post the fifth scaffold

**Figure 6:**
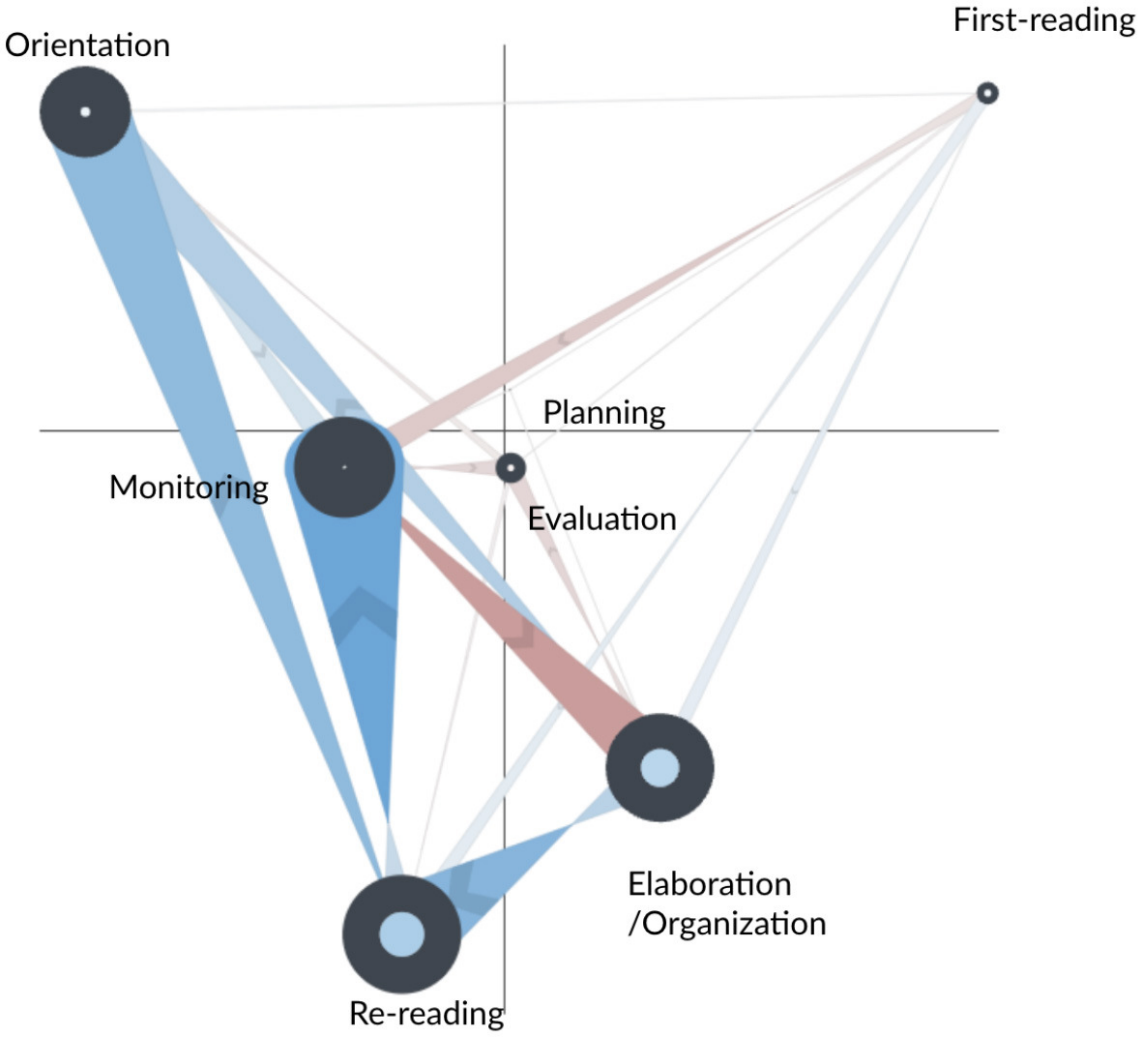
Subtracted ONA network of the SRL process between the control (CN, in red) and adaptive scaffolding (AS, in blue) groups during the fifth segment of the study task.

Figure 6 shows the network subtraction for the CN and AS learners at the end of the fifth segment (CN in read; AS in blue). In the fourth segment, the results suggested that AS learners were engaged in a process of re-reading to gather useful information for writing, as indicated by the transition from re-reading to elaboration/organization in Figure 5. However, after receiving the fifth scaffold, the direction of transition reversed (from elaboration/organization to re-reading), suggesting that learners began to check their writing by referring back to previously read pages. Moreover, AS learners made stronger transitions from re-reading to orientation and from re-reading to monitoring, which were not predominant in the fourth segment. This suggests that, after receiving the fifth scaffold, which advised learners to “revise your writing based on the task instruction and marking rubric”, AS learners followed the suggestions and initiated a process of finalizing, revising, and refining their essay by referring back to the task instructions and rubric, as well as their notes.

In contrast, CN learners seemed to ramp up their writing activities as they neared the end of the task with time running short. This is evidenced by a stronger transition from monitoring to elaboration/organization, suggesting they became more actively engaged in writing after revisiting previously made annotations and keeping a close eye on the remaining time. It appears that the time pressure acted as a spur to their shift into more intensive monitored writing, suggesting that their activities were largely driven by time constraints as opposed to a systematic or methodical approach to learning exhibited by AS learners.

#### 3.2.3. Comparing AS and FS groups post the fourth scaffold

**Figure 7:**
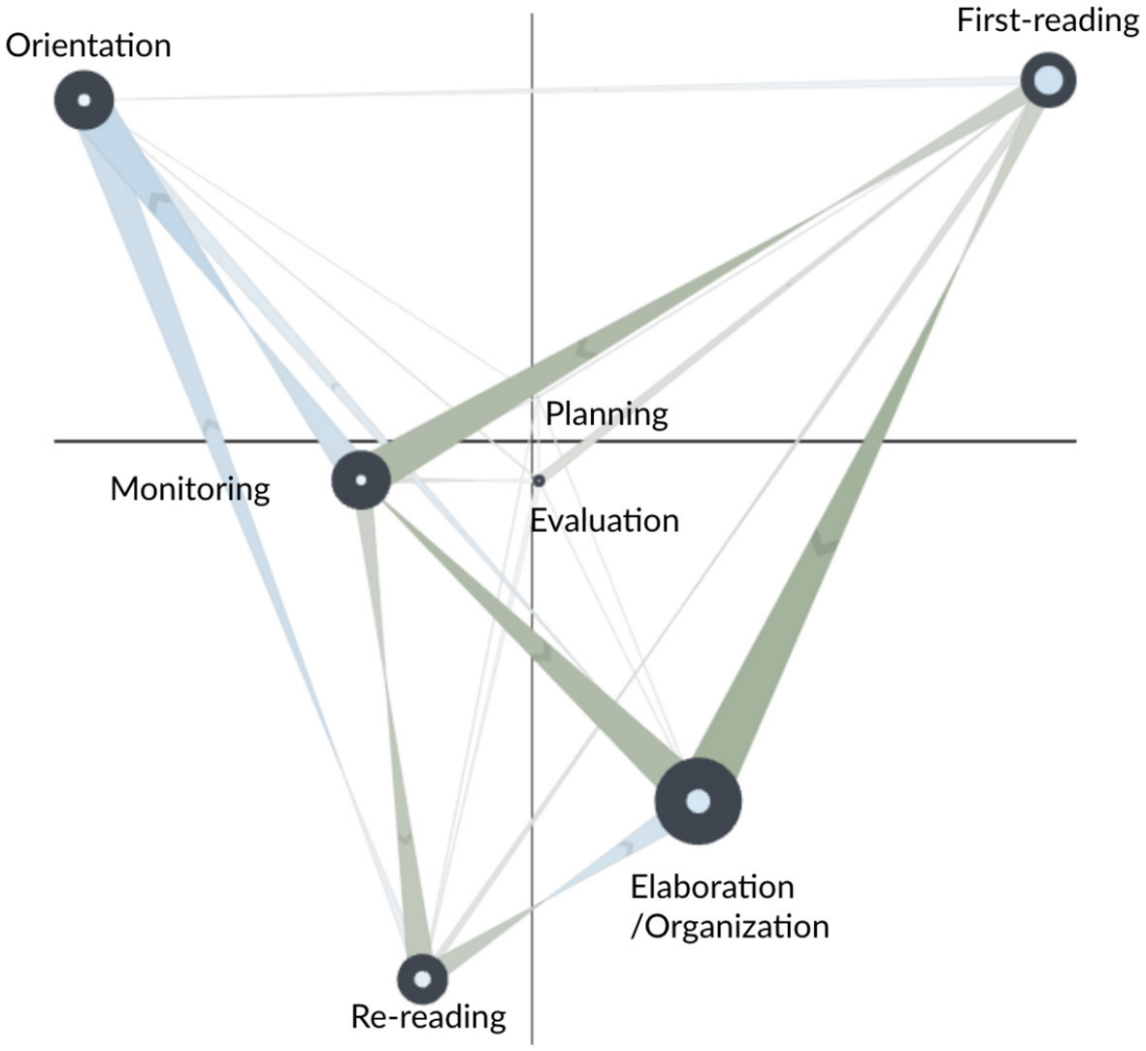
Subtracted ONA network of the SRL process between the fixed scaffolding (FS, in green) and adaptive scaffolding (AS, in blue) groups during the fourth segment of the study task.

Figure 7 shows the network subtraction for the FS and AS learners at the end of the fourth segment (FS in green; AS in blue). Differences between the two groups are highly similar to the differences we observed between the CN and AS learners in the fourth segment. In particular, the stronger transitions for the FS learners were from first-reading to monitoring, from first-reading to elaboration/organization, from monitoring to elaboration/organization, and from monitoring to re-reading. These transitions suggest that the FS learners were actively monitoring their reading and re-reading processes, utilizing the information gleaned from reading to inform their writing, and subsequently monitoring their writing process. On the contrary, the AS learners made more frequent transitions from re-reading to orientation, from monitoring to orientation, and from elaboration/organization to orientation. These transitions suggest that the AS learners' activities were primarily guided by an understanding of the task requirements before embarking on reading and writing tasks.

#### 3.2.4. Comparing AS and FS groups post the fifth scaffold

**Figure 8:**
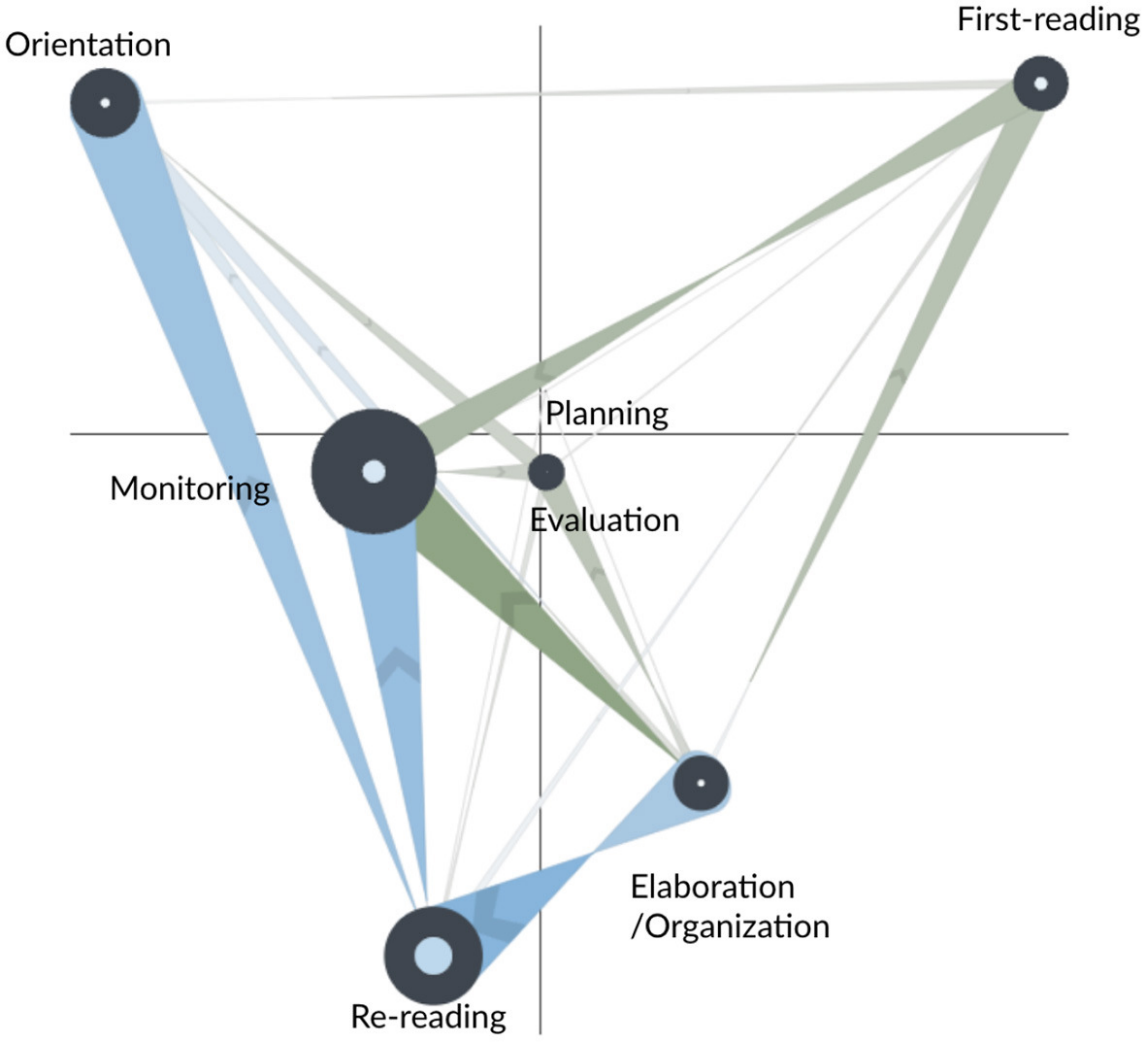
Subtracted ONA network of the SRL process between the fixed scaffolding (FS, in green) and adaptive scaffolding (AS, in blue) groups during the fifth segment of the study task.

Figure 8 shows the network subtraction for the FS and AS learners at the end of the fifth segment (FS in green; AS in blue). AS learners made more frequent transitions from re-reading to orientation, from re-reading to monitoring, and from elaboration/organization to re-reading. Additionally, AS learners made more frequent self-transitions to re-reading. Together, these transitions suggest that AS learners were more engaged in a process of checking and refining their essay after receiving the fifth scaffold. Conversely, FS learners made more frequent transitions from first-reading to monitoring, from elaboration/organization to monitoring, and from elaboration/organization to first-reading. These transitions suggest that FS learners were engaged in writing while still reading new information (pages that were not previously read).

The analysis of SRL process patterns for the fifth segment suggests parallels between AS-FS and AS-CN learner comparisons. AS learners, across both comparisons, showed a transitions-related essay refinement and revision processes—as indicated by more frequent transitions to elaboration/organization, which capture writing behaviors via keystrokes—while their counterparts, both FS and CN learners, primarily engaged in reading and writing activities, along with monitoring their task progress. This result to a consistent divergence in SRL processes between AS learners and the other two groups during the fifth segment.

## 4. Discussion

This study used the ONA technique to model learners' SRL processes at both task level and segmented level. By comparing learners across different scaffolding groups, a number of intriguing findings were revealed.

### 4.1. Research question 1: effectiveness of scaffolding at the task level

This study examined the extent to which the existence of scaffolding facilitates effective SRL processes by comparing the ONA visualizations between AS and CN learners at the overall task level, and the differences in the second dimension of ONA are found to be statistically significant. It is found that AS learners were primarily engaged in task-guided reading and writing, while CN learners were predominantly focused on reading and writing while monitoring their learning progress. Meanwhile, the ONA comparison is also conducted between AS and FS learners to examine how the adaptivity of scaffolding may affect the effectiveness of scaffolding in promoting SRL processes. The comparison of SRL processes between AS and FS learners yielded analogous visualizations to those observed between AS and CN learners, and the differences in the second dimension of ONA are found to be statistically significant. Similar to the CN learners when compared to the AS learners, FS learners exhibited a learning approach that emphasized reading and writing while intermittently monitoring their learning progress. This result parallels the ONA visualizations between AS and CN learners, further corroborating the finding that learners without adaptive scaffolding tend to involve themselves more intensely in the reading and writing processes, monitoring their learning progress as necessary.

The SRL processes exhibited by AS learners present a fitting illustration of the key components of Winne and Hadwin's COPES model (Winne and Hadwin, 1998; Winne, 2018). As indicated by the COPES model, SRL learners strategically select learning tactics based on the specific conditions of the learning environment and meticulously align these chosen tactics to fulfill task requirements (Winne and Hadwin, 1998; Winne, 2017; Fan et al., 2021a). Based on our findings at the task level, AS learners demonstrated these abilities prominently. They showcased a strategic approach in their learning processes, with a clear focus on understanding the requirements of the task before delving into their reading and writing activities. This behavior signifies a mindful and well-planned approach to learning that aligns with the theoretical tenets of a self-regulated learner. Hence, given the strategic SRL processes demonstrated by the AS learners, it can be inferred that the provision of adaptive scaffolding aligns with the promotion of strategic SRL processes.

Drawing upon the findings, it can be inferred that AS learners' deliberate choice of what to read and write, as well as their ongoing integration of task instructions, underscores a heightened level of metacognitive engagement in their learning process. As Butcher and Sumner (2011) concluded, metacognitive processes in an essay-writing task mainly involve three activities—critical analysis on existing representation (e.g., reading material and the essay constructed by the learners), active searching for relevant information from reading material, and active revising on the existing representation. In the current study, the task-guided SRL processes which were performed by AS learners were highly consistent with those metacognitive processes as posited in Butcher and Sumner (2011). AS learners' keen engagement in task orientation and reading resonates with Butcher and Sumner (2011)'s emphasis on the critical analysis of existing representations as a key metacognitive process in essay-writing. Furthermore, their pattern of revisiting previously read information, alongside reading new material, and subsequently organizing their essays aligns with the active search for relevant information and the proactive revision of existing representations, further emphasizing their metacognitive engagement in the task.

### 4.2. Research question 2: effectiveness of scaffolding at different learning segments

To address RQ2, this study carried out a segmentation analysis, aiming to uncover the extent to which each individual scaffold is associated with different SRL processes across different scaffolding groups. This approach is intended to offer nuanced insights into how immediate adjustments in SRL processes correspond with different scaffolding conditions.

The fourth scaffolding encouraged learners to not only start writing but also to write strategically by using various SRL tactics (e.g., as evidenced from the message in the fourth scaffolding, to review annotations, to check requirements, or to read selectively). The findings showed that the FS and CN learners primarily engaged in a more linear, reading-centric process, continuously progressing page-by-page through the reading material and extracting information for their essays. In contrast, the AS learners demonstrated a more strategic approach to writing, regularly referring back to previously read pages or annotations. In sum, compared to those who did not receive scaffolding and those who only received fixed scaffolding, the learners who received adaptive SRL scaffolding tended to engage in more strategic writing and reading processes. While the differences we observed at the segment level were not statistically significant—likely due to low power—they align with previous studies which found that scaffolding—especially adaptive scaffolding—is effective at encouraging strategic learning processes (Azevedo et al., 2004). Furthermore, given that ‘orientation' is classified as a metacognitive process according to the SRL model proposed by Bannert (2007), the current study's findings highlight that adaptive SRL scaffolding, when compared to control conditions and fixed scaffolding is more potent in fostering metacognitive learning processes (Sonnenberg and Bannert, 2016).

From the fourth segment to the fifth segment, we observed different SRL process transitions among different scaffolding groups. Specifically, in the comparison between the AS and CN groups, it was observed that those in the CN group failed to exhibit certain SRL processes, including orientation, monitoring, and re-reading. This lack of guidance may have led them to allocate an excessive amount of time to reading, consequently leaving insufficient time for writing and minimizing the opportunities to review and revise their written article. From the theoretical perspective, this can be explained by the phenomena of availability deficiency, which happens when a learner does not have the knowledge or is unaware of the available cognitive or metacognitive processes that can be used in learning (Veenman et al., 2006; Wirth, 2009). On the contrary, as AS learners exhibited a multitude of SRL processes , suggesting that the implementation of SRL scaffolding may benefit learners by making them aware of available SRL processes. Meanwhile, compared to the FS learners, AS learners still demonstrated earlier SRL processes in task-guided writing and revising their essays. This concludes that, despite the fact that learners in both AS and FS groups received scaffolding which made them being aware of available SRL processes, implementing adaptive and fixed scaffolding still led to different SRL patterns. The potential reason for this finding might be that fixed scaffolding is unable to address the unique needs of individual learners, which could lead to the noncompliance to the provided scaffolding (Guo, 2022). Hence, learners are more likely to be receptive to scaffolding when the content is tailored to meet their particular SRL needs. From a theoretical standpoint, a scenario where learners are aware of the existence of various SRL processes, yet refrain from actively utilizing them, aligns with what is typically referred to as ‘production deficiency' (a situation where a learner who is aware of certain learning tactics but failed to utilize them) (Winne, 1997; Veenman et al., 2006; Wirth, 2009). This can be observed in the ONA models in comparison between AS and FS learners, which revealed that although both AS and FS learners received scaffolding, they nevertheless demonstrated distinct SRL processes. Thus, it could be surmised that the adaptivity inherent in scaffolding might play a crucial role in mitigating the phenomenon of production deficiency, thereby enhancing the effectiveness of promoting SRL processes. Overall, our findings lend support to the premise that adaptive scaffolding is potentially the most advantageous approach to support learners' SRL, by fostering an awareness of available SRL resources and concurrently encouraging early utilization of SRL processes.

## 5. Conclusion

In conclusion, this study utilized the ONA technique to explore varying SRL processes among higher education students participating in a two-hour reading and writing task under three different conditions: no scaffolding, fixed scaffolding, and adaptive scaffolding. Moreover, our investigation extended to both the overall task level and segmented levels. Findings illuminated the profound influence of adaptive scaffolding in fostering learners to be more task-oriented and metacognitively engaged, thus enabling more effective and strategic reading and writing processes. Conversely, learners under fixed scaffolding and no scaffolding conditions tended to delve more into the reading and writing processes, while concurrently monitoring their progress. These findings highlight the potential benefits of incorporating adaptive scaffolding in the learning context to bolster learners' self-regulation.

### 5.1. Research implication and future practice

At least two research implications and one practical implication can be concluded from our study. First, this study focused on the effectiveness of scaffolding using segmentation to analyze the immediate adjustments in SRL processes after the introduction of each scaffold by segmenting the learning task according to when the scaffolding was provided. Thus, segmentation analysis allowed an in-depth and detailed analysis of each scaffold. Future research should continue using segmentation analysis to deepen the understanding of learners' SRL process and the effectiveness of scaffolding at a segmented level. Second, this study offers the first insights of using the ONA technique to model learners' SRL processes in relation to scaffolding. Compared to other widely-adopted analytical techniques in understanding learners' SRL processes and the effects of scaffolding (e.g., process mining), the ONA technique is advantageous to the extent that it can address four dimensions of learning processes at once, including frequency, continuity, sequentiality, and role of actions (i.e., the function or functions that a learning action plays, which can be different in different learning contexts), which are aspects that other predominately-used techniques alone cannot (Fan et al., 2023). Moreover, ONA's deterministic node position layout supports the creation of subtracted networks to visualize differences in SRL processes between groups of learners. As such, future studies are recommended to continue in utilizing the ONA technique to model learners' SRL. For example, a promising direction could be modeling learners' use of learning tactics (e.g., highlighting) by using ONA and exploring if different transitions among learning tactics visualized on ONA can inform different learning strategy patterns. Moreover, we successfully identified the manifestation of distinct SRL processes in learners under different scaffolding conditions. An intriguing direction for future studies would be to probe whether these SRL processes, and the extent to which they are employed, correlate with variations in learning performance. This could yield a deeper understanding of the extent to which adaptive scaffolding could promote learning outcomes. Lastly, the results of this study may also provide some suggestions for practical and instructional improvement. Because we found that adaptive scaffolding can be effective in mitigating the phenomena of availability and production deficiency by not only making learners aware of available SRL resources but also promoting early SRL actions, educational instructors can take advantage of this positive effect by embedding adaptive scaffolding within the learning task. In addition, because we found that adaptive scaffolding was more closely related to more task-guided SRL processes, future instruction can leverage this advantage to design more adaptive scaffolding to further support the development of SRL.

### 5.2. Limitations

Our study has several limitations. The primary limitation constraint stems from the time limit set for the written task. With a 120-minute time limit, learners might have experienced pressure to complete the task, potentially amplifying the observed differences between the AS and FS conditions. However, we must bear in mind that this was not a tightly controlled laboratory study. Instead, it took place in a classroom setting, adhering to authentic course requirements. Future studies could address this limitation by allotting more ample time for task completion, which could help minimize the potential impact on the disparities between the FS and AS conditions. Second, in order to improve the readability of the ONA visualizations, the edge weights of each network were scaled up by using a consistent multiplier. While this maintains the relative differences between the examined connections, readers may perceive the differences as larger than they were. Moreover, our study did not find significant differences among the various scaffolding groups in terms of their SRL processes at the segmented level. However, our power analyses suggest that the lack of statistical significance for some comparisons may be due to a low N. A possible explanation for the small differences observed in some comparisons is the relatively short duration for each segment. Hence, it may be difficult for learners to significantly adjust their SRL processes just within a short period of time and encouraging more effective SRL processes should be proposed and implemented as a long-term process. We might expect the differences we observed to be more prominent for a longer learning task or a study with more participants. Relatedly, our ONA analysis only explored transitions between pairs of codes. Stronger differences may be observed for longer sequences, but this approach could reduce the interpretability of the results (Swiecki et al., 2019). Third, SRL is inherently contextual (Winne, 2010), and therefore, the research findings in the current study can only be referred to other similar learning tasks (i.e., read-and-write essay-writing tasks). As such, we suggest future studies investigating SRL learning processes in different learning contexts to test the generalizability of our findings. Lastly, our study encountered some technical difficulties that led to the exclusion of some participants from the data analysis, as illustrated in Table 1. This resulted in varying attrition rates across the different scaffolding groups. We recommend that future studies aim to replicate our research to verify the repeatability of our results.

Despite these limitations, our results suggest that adaptive scaffolds are associated with positive changes in SRL processes compared to providing fixed scaffolds or no scaffolds at all. Specifically, we found that adaptive scaffolds are effective at 1) encouraging learners to adopt metacognitively task-guided SRL processes and 2) bringing awareness of and facilitating early engagement in SRL processes. This study demonstrates significant novelty in not only deepening our understanding of the effects of scaffolding at the segmented task level but also in using a contemporary network analytic technique to evaluate the effects of adaptive scaffolding on learners' SRL processes.

## Data availability statement

The raw data supporting the conclusions of this article will be made available by the authors, without undue reservation.

## Ethics statement

The studies involving human participants were reviewed and approved by the Ethical Committee in the University of Chinese Academy of Sciences approved the present research. The patients/participants provided their written informed consent to participate in this study.

## Author contributions

TL wrote and revised the draft of the manuscript. YF contributed to the conception and design of the study. YT, YW, and BY performed the ordered network analysis for all research questions. SS, XL, MR, JV, LL, IM, MB, and JM designed the learning platform from which data is collected for this study. ZS, Y-ST, DS, and DG provided guidance on the overall design and data analysis processes. All authors contributed to manuscript revision, read, and approved for submitted version.
